# On the Brain of a Crustacean: A Morphological Analysis of CaMKII Expression and Its Relation to Sensory and Motor Pathways

**DOI:** 10.1371/journal.pone.0064855

**Published:** 2013-05-31

**Authors:** Dib Ammar, Evelise M. Nazari, Yara M. R. Müller, Silvana Allodi

**Affiliations:** 1 Programa de Pós-Graduação em Biologia Celular e do Desenvolvimento, Departamento de Biologia Celular, Embriologia e Genética, Centro de Ciências Biológicas, Universidade Federal de Santa Catarina, Santa Catarina, Brazil; 2 Programa de Pós-Graduação em Morfologia, Instituto de Ciências Biomédicas, Universidade Federal do Rio de Janeiro, Rio de Janeiro, Brazil; 3 Programa de Pós-Graduação em Ciências Biológicas - Fisiologia, Instituto de Biofísica Carlos Chagas Filho, Universidade Federal do Rio de Janeiro, Rio de Janeiro, Brazil; CNRS, France

## Abstract

Calcium/calmodulin kinase II (CaMKII) is a Ca^2+^-activated enzyme that is abundant in vertebrate and invertebrate brains. However, its characterization is poorly addressed in the nervous system of crustaceans, and, to our knowledge, no studies have determined the microanatomical location of CaMKII in a crustacean species. In this study, we found labeling of CaMKII in the eyestalk and brain of the prawn *Macrobrachium acanthurus*, by means of immunohistochemistry and Western blotting. Antibodies against neuron (ß tubulin III), glutamate receptor (GluA1), and FMRFamide were used in order to further characterize the CaMKII-labeled cells in the brain. In the eyestalk, strong labeling with CaMKII was observed in the photoreceptors. These cells, especially in the rhabdom, were also reactive to anti-ß tubulin III, whereas the pigment cells were labeled with anti-CaMKII. GluA1 co-located with CaMKII in the photoreceptors. Also, CaMKII appeared in the same sites as FMRFamide in the deutocerebrum, including the olfactory lobe, and in the tritocerebrum, specifically in the antennular neuropil, indicating that the synaptic areas in these regions may be related to sensory-motor processing. In the brain, the identification of cells and regions that express CaMKII contributes to the understanding of the processing of neural connections and the modulating role of CaMKII in decapod crustaceans.

## Introduction

Calcium (Ca^2+^) plays a dual role within nerve cells. It carries electrical current through voltage-gated channels in the membrane, and it also acts as a second messenger by activating a wide range of intracellular proteins [1], [2], [3]. Calcium/calmodulin-dependent protein kinase II (CaMKII) is a Ca^2+^-activated enzyme that is highly abundant in vertebrate and invertebrate brains [4], [5], [6].

The biochemical and/or molecular characterization of CaMKII in the nervous system of some invertebrate groups has been carried out mainly in *Caenorhabditis elegans* [5], *Drosophila melanogaster* [7], *Manduca sexta* [8], and *Aplysia californica* [2]. Few studies have addressed the presence, as well as the physiological and biochemical characterization of CaMKII in the nervous system of crustaceans [9], [10], [11], [12]. This lacuna in studies of crustacean CaMKII needs to be filled, since crustaceans have a complex nervous system that has been used as a model for research in neurobiology.

In vertebrates, four homologous CaMKII isoforms (α, β, γ, δ) are encoded by separate genes, and alternative splicing in their variable linker domain provides additional diversity [3], [13]. There are differences in the tissue distribution of the isozymes; for instance, in mammals, α is most concentrated in the forebrain and β in the cerebellum [14], [15]. In invertebrates, few studies have examined the CaMKII encoding genes, and only in *Drosophila.* In this species, [15] and [16] showed that CaMKII is encoded by a single gene with up to 18 isoforms, which are generated by alternative splicing.

Knowledge of the structural features of the decapod crustacean brain may facilitate the investigation of the basic principles of a functional nervous system. Anatomically, the crustacean brain is constituted by the fusion of the first three ganglia of the ventral nerve cord: the protocerebrum in the anterior region of the brain, the deutocerebrum in the middle region, and the tritocerebrum in the posterior region [17], [18], [19]. The basic functions of the crustacean brain are concerned with the activity of the photoreceptive cells located in the compound eyes, linked to the protocerebrum, and also with the activity of the chemoreceptive and mechanoreceptive sensilla located on the antennules and antennas, linked to the deutocerebrum and tritocerebrum respectively [20], [21], [22]. In a previous study [19], we reported subtle differences in the brain microanatomy of freshwater prawns and marine shrimps, mainly in the olfactory lobes (OL). In the present study, we examined the *in situ* expression of CaMKII, and its relationship to other neurotransmitters or neuromodulators, in the eyestalk and in the brain of the freshwater prawn *Macrobrachium acanthurus,* in order to expand knowledge of the interaction of sensory and motor inputs in invertebrates.

## Materials and Methods

### Animals

Eighteen adult males of the freshwater palaemonid prawn *Macrobrachium acanthurus* (body length 80–170 mm; mean body length 110.81 mm, standard deviation 27.61 mm) were captured in streams in Florianópolis, state of Santa Catarina, Brazil. The prawns were transferred to the laboratory and placed in 60 L water tanks at 25°C (±1), under constant aeration and the natural light:dark cycle. The prawns were fed once a day with commercial pellet food (Alcon Bottom Fish®). After two weeks, the brains were analyzed. All procedures used during this study were performed after approval by the Brazilian National Environmental Committee (IBAMA certificate number 15294-1/2008), and every effort was made to minimize animal suffering.

### Histology

Twenty prawns were cooled on ice for 10 min prior to dissection. They were then decapitated, and the dissected brains were fixed with 4% formaldehyde, freshly prepared from paraformaldehyde, in 0.1 M phosphate-buffered saline (PBS) for 4 h and then washed in 0.1 M PBS. The brains were embedded in Paraplast® and serially sectioned at 7 µm in the horizontal plane (60 to 70 sections for each brain). The sections were mounted on gelatin-coated slides. One series of sections was dewaxed, hydrated, and stained with Mallory’s trichrome for routine histological observation.

### Immunohistochemistry

Series of sections destined for the immunohistochemical reactions were dewaxed, hydrated, and then washed in PBS with 0.3% Triton X-100, incubated with 5% normal goat serum, and then with the primary antibody overnight at 4°C. To immunodetect the CaMKII, we used the primary antibody rabbit anti-CaMKII diluted 1∶100. It was developed from the nervous system of the lobster *Panulirus interruptus* (antibody kindly made available by Dr. Michele Withers, Volen Center and Biology Department, Brandeis University), and was identified from the partial cDNA clone from a lobster that has a variable domain analogous to that seen in mammalian and *Drosophila* CaMKII [11]. Different primary antibodies were used to assess the identity of the reactive CaMKII cells as neurons: rabbit anti-ß tubulin III (Abcam), diluted 1∶100; mouse anti-glutamate receptor (GluA1; Dako), diluted 1∶100, to label chemosensory neurons [23]; and rabbit anti-FMRFamide (Sigma), diluted 1∶100, to label neurites [24], [25], [26].

Next, the sections were washed again in PBS with 0.1% Tween 20 and incubated with the secondary antibody (fluorescein or peroxidase anti-rabbit IgG and rhodamine anti-mouse IgG; Sigma) for 2 h before a rinse with 0.1 M PBS. A 0.9% sodium chloride solution was used to wash the sections before they were incubated with a solution of diaminobenzidine (DAB; Sigma). Finally, the sections were mounted with Entellan (Merck) or Gel Mount (Biømeda), then viewed and photographed using either an Olympus light microscope or a Zeiss confocal microscope (LSM 510 software). The confocal laser-scanning microscope images were a composite of 14 optical sections at 500 nm intervals. To observe CaMKII or GluA1 (fluorescein: green) and anti-ß tubulin III (rhodamine: red), excitation with the 488 nm laser (filter: BP 505–550 nm) and 543 nm laser (filter: LP 570 nm) was used, respectively. For the negative control sections, the same procedure was followed, except that the primary antibody was omitted.

### Brain Extract Preparation

Brains were homogenized on ice in PBS (0.1 M, pH 7.4) with a protease inhibitor cocktail (1 mM PMSF, 1 mM benzamidine, and 100 mM caproic acid). The homogenates were centrifuged for 30 min at 10,000 g at 4°C, twice. The precipitate was discarded, and the supernatant was separated and stored at −186°C until analysis. Total protein in each sample was estimated by the method of Lowry as modified by [27].

### SDS-PAGE and Western Blotting

Extracts from the brains were separated by 10% SDS-PAGE. First, the samples were dissolved in treatment buffer (1 M Tris-HCl pH 6.8, 20% glycerol, 20% SDS, 0.2% bromophenol blue, 5% β-mercaptoethanol) and boiled for 2 min. The brain extract was applied to 10% separating gels with 3% stacking gels, and the proteins were separated at 120 V for 3 h in 0.05 M Tris glycine-buffered solution with 0.1% SDS, pH 8.3. The proteins were observed after staining with Coomassie brilliant blue R250. The molecular weights were determined with a kit containing mid-range protein molecular weight standards from 200 kDa to 14.3 kDa (Sigma). Western blots from SDS-PAGE were carried out at 400 mA for 1 h at 4°C. The proteins were transferred to a nitrocellulose membrane in transfer buffer (cyclohexyl-3-aminopropanesulfonic) with 10% methanol (pH 11.0). The membrane was blocked overnight at 4°C with skim milk and rinsed with TBS for 5 min. Then, it was incubated with rabbit anti-CaMKII, diluted 1∶2500 with TBS, for 1 h at room temperature on a shaker. The membrane was rinsed five times, 5 min each, with TBS and incubated for 1 h with alkaline phosphatase-conjugated goat anti-rabbit IgG, diluted 1∶1000 with TBS. The color reaction was developed with nitro blue tetrazolium/5-bromo-4-chloro-3-indolyl-phosphate (NBT/BCIP) in a dark chamber.

## Results

### Morphology

**Figure 1:**
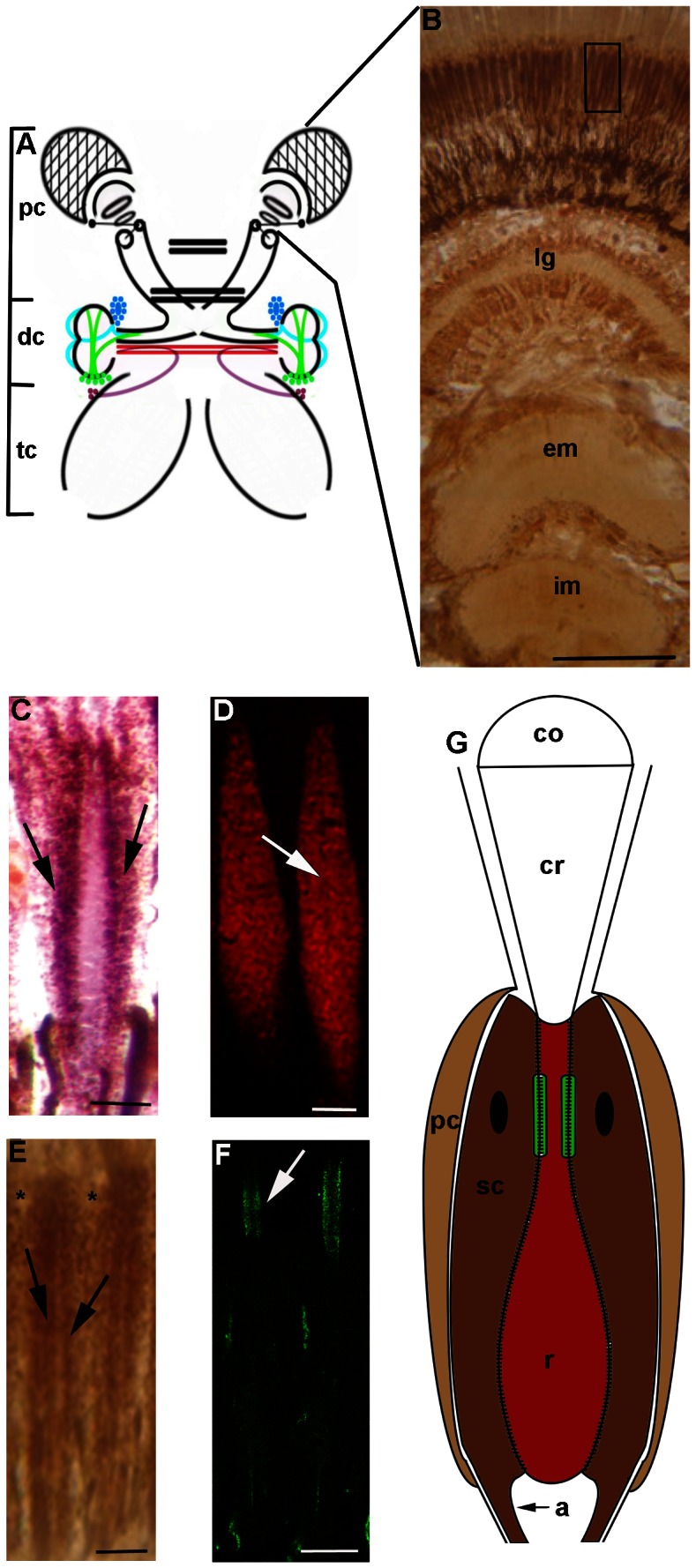
Brain of *Macrobrachium acanthurus*. **A:** Diagram showing the eyestalk (magnified histological section in B), protocerebrum (pc), deutocerebrum (dc), and tritocerebrum (tc). **B:** Low magnification of a longitudinal section of the eyestalk, stained with anti-CaMKII, showing the compound eyes and the optic neuropils: *lamina ganglionaris* (la), external medulla (em), and internal medulla (im). The black rectangle in the retina encompasses a few ommatidia, which are shown in C–F. **C:** Longitudinal section showing photoreceptors (arrows) stained with Mallory’s trichrome. **D:** Rhabdoms (arrow) labeled with anti-ß tubulin III. **E:** Label for anti-CaMKII antibody in photoreceptors (arrows) and pigment cells (asterisks). **F:** Ommatidia labeled with anti-GluA1 (arrow). **G**: Diagram showing the structure of an ommatidium. Cornea (co), crystalline cone (cr), sensory cell (sc), rabdom (r), pigment cell (pc) and axon (a). The colors in the diagram represent the colors of labeling in C–E. Bars A 100 µm, B–F 7 µm.

In the present study, we immunolocated the CaMKII in the sensory and associated regions of the eyestalk, medial protocerebrum, deutocerebrum (including the OL), and the tritocerebrum of the freshwater prawn *M*. *acanthurus.* A diagram of the whole brain including the eyestalk is depicted in Figure 1A.

### Eyestalk

Figure 1B shows an overall view of a longitudinal histological section of the eyestalk, stained with the antibody against CaMKII, where the retina and the optic neuropils can be distinguished. Figure 1C shows a higher magnification of the retina, illustrating the components of some ommatidia as evidenced by Mallory trichromic staining. Photoreceptors (sensory neurons), especially the rhabdom, were reactive to anti-ß tubulin III (Figure 1D). CaMKII immunohistochemistry revealed strong labeling in the sensory neurons (Figure 1E) and weaker labeling in the adjacent pigment cells. In addition, anti-GluA1 labeled the rhabdom (Figure 1F). Figure 1G shows a diagram of an ommatidium.

### Brain (Medial Protocerebrum, Deutocerebrum and Tritocerebrum)

**Figure 2:**
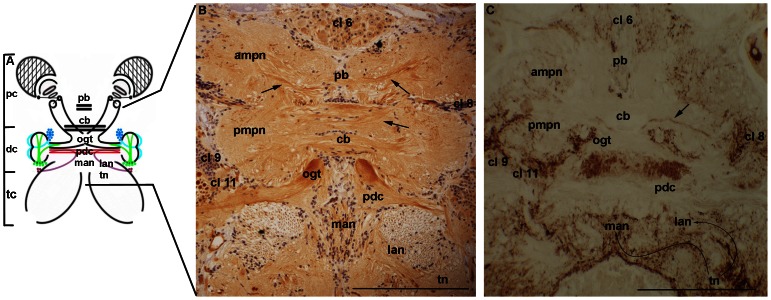
Frontal section of the median protocerebrum, deutocerebrum, and tritocerebrum of *Macrobrachium acanthurus.* **A:** Diagram showing the brain regions, emphasizing the structures in the histological sections. **B:** Immunoreactivity to CaMKII. **C:** Immunoreactivity to FMRFamide. Anterior medial protocerebral neuropil (ampn), posterior medial protocerebral neuropil (pmpn), protocerebral bridge (pb), protocerebral tracts (arrows), central body (cb), olfactory-globular tract (ogt), prospective deutocerebral commissure (pdc), medial antennular neuropil (man), lateral antennular neuropil (lan), tegumentary neuropil (tn), cluster 6 (cl 6), cluster 8 (cl 8), cluster 9 (cl 9), and cluster 11 (cl 11). Bars 100 µm.

A diagram of the whole brain (Figure 2A) illustrates the location of the median protocerebrum in the whole brain. Immunohistochemistry revealing CaMKII showed intense labeling of a bundle of fibers within the protocerebral bridge, central body, and protocerebral tracts in the anterior medial protocerebrum and in the posterior medial protocerebrum. In the deutocerebrum, an intense CaMKII immunoreaction was observed in the olfactory-globular tract (OGT) and in the prospective deutocerebral commissure, as also observed by [19] (Figure 2B). The immune reaction using the FMRFamide antibody showed labeled cells in the cell clusters 6, 8, 9, and 11. Additionally, FMRFamide-immunoreactive fibers that link the tegumentary neuropils, which are located bilaterally in the tritocerebrum, to the medial antennular neuropils and to the lateral antennular neuropils were observed (Figure 2C).

**Figure 3:**
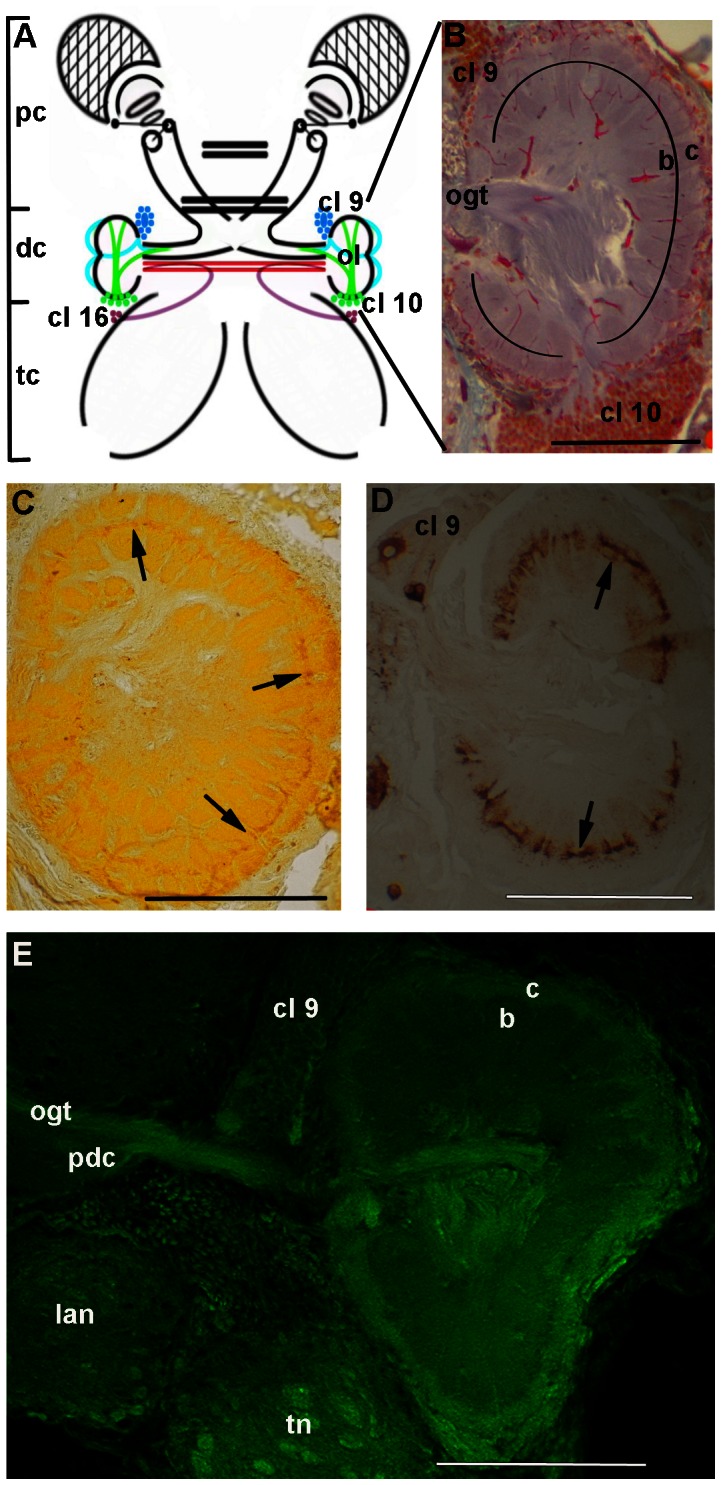
Olfactory lobe of *Macrobrachium acanthurus*. **A:** Diagram showing the protocerebrum (pc), deutocerebrum (dc), tritocerebrum (tc), and in more detail, the olfactory lobes (ol), and clusters 9 (cl 9), 10 (cl 10) and 16 (cl 10). **B:** Frontal section of the olfactory lobe stained with Mallory’s trichrome, showing the glomeruli and their regions: cap (c), subcap (black line), base (b), and clusters 9 (cl 9) and 10 (cl 10). **C:** Olfactory lobe showing the reactivity to anti-CaMKII (arrows) in the subcap region. **D:** Olfactory lobe showing the reactivity to anti-FMRFamide (arrows). **E:** Confocal image of a part of the deutocerebrum, showing the reactivity to anti-CaMKII in the olfactory-globular tract. Olfactory-globular tract (ogt), prospective deutocerebral commissure (pdc), lateral antennular neuropil (lan), tegumentary neuropil (tn), cluster 9 (cl 9), cap (c), base (b). Bars 100 µm.

The OL are the most prominent neuropils of the deutocerebrum, and are located in the more lateral region of the cerebral ganglion (Figure 3A). The cortical region of the OL is constituted by juxtaposed glomeruli (Figure 3B). The medullar region of the OL is composed of the neurites from the interneurons constituting cluster 9, and neurites from projection neurons constituting cluster 10. Each glomerulus is divided into three areas (as seen in Figure 3B), and all were labeled with CaMKII (Figure 3C): the cap, which receives sensory stimuli from the antennules and antennae [20], [26]; the base, which receives sensory stimuli through the OGT [22], [28]; and the subcap, which is located between the cap and the base. Our results showed that the subcap was also labeled with both the anti-CaMKII (Figure 3C) and the anti-FMRFamide (Figure 3D). Figure 3E shows a confocal image of a part of the deutocerebrum, reacted for CaMKII.

**Figure 4:**
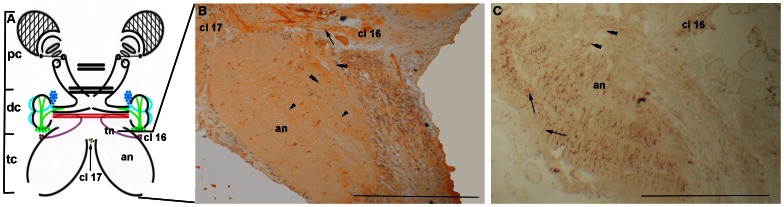
The antennal neuropil of *Macrobrachium acanthurus.* **A:** Diagram of the prawn brain showing the location of the antennal neuropil (an), the tegumentary neuropil (tn), and clusters 16 (cl 16) and 17 (cl 17) in the tritocerebrum. **B:** Frontal section of the antennal neuropil (an), showing the reactivity to anti-CaMKII. **C:** Immunoreactivity to FMRFamide. Cluster 16 (cl 16), cluster 17 (cl 17), motor neuron axon (arrows), sensory neurons of the antenna (arrowheads), and sensory neurons (double arrowheads). Bars 100 µm.

The tritocerebrum (see the diagrams in Figures 2A and 4A) showed a large tegumentary neuropil (Figure 2B and C). The tegumentary nerves, labeled with both CaMKII and FMRFamide, run toward the median line, projecting to different parts of the medial antennular neuropil and the lateral antennular neuropil (Figure 2B and C). The antennal neuropil displayed sets of fibers immunoreactive to CaMKII, which run from the sensory neurons of the antenna, traversing longitudinally the antennal neuropil (Figure 4B) to the deutocerebrum and median protocerebrum. The projections of the neurons located in the lateral margins of the tritocerebrum cross the antennal neuropil transversely and join the fibers that run from the sensory neurons of the antenna to the OL and the OGT. The immune assay against FMRFamide showed a network connecting the sensory neurons of different tritocerebrum cell clusters to the antennal neuropil (Figure 4C).

### Western Blotting

**Figure 5:**
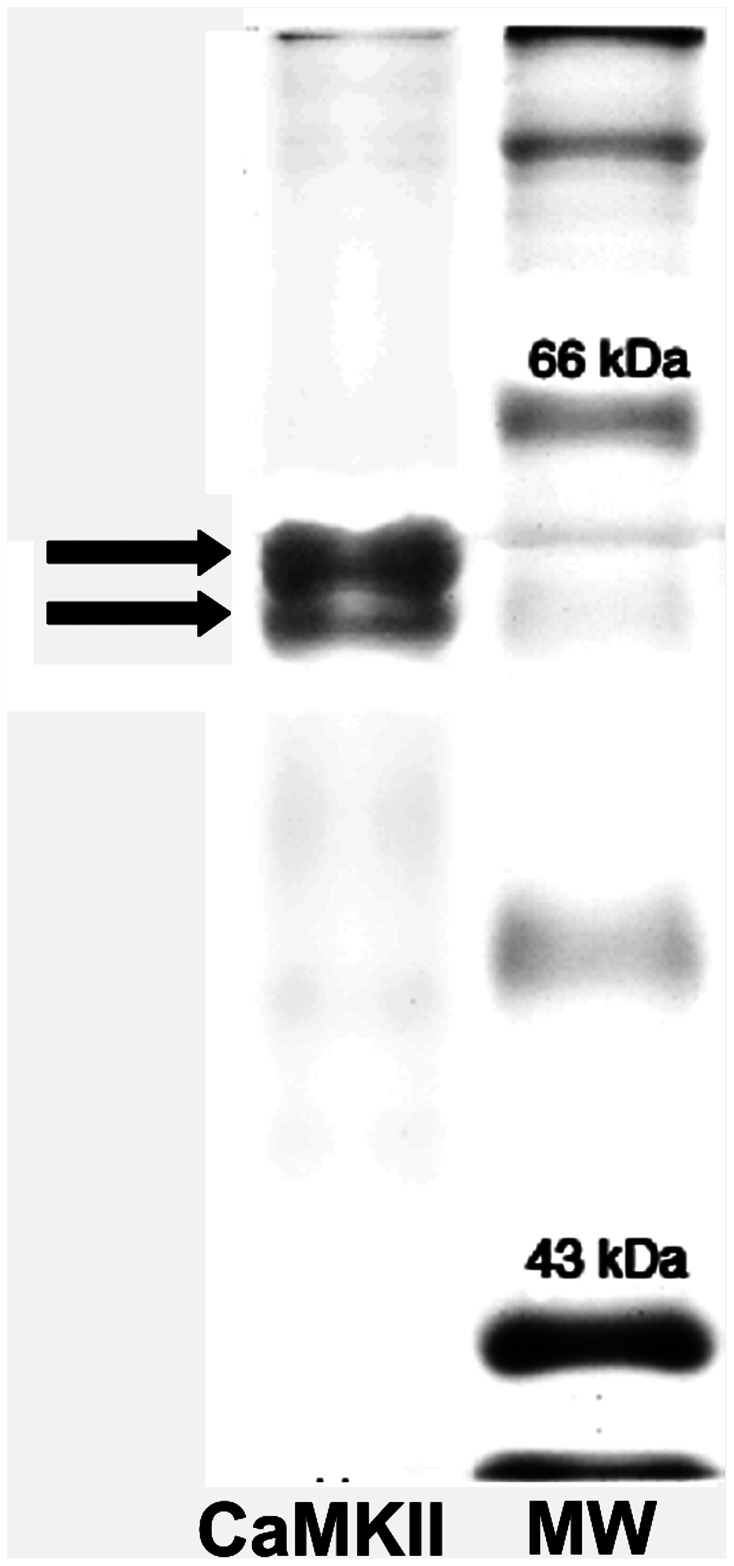
Western blotting of *Macrobrachium acanthurus* brain, showing the presence of two adjacent 60 kDa bands of CaMKII.

In order to confirm the presence of CaMKII, homogenates of *M. acanthurus* brain displayed the CaMKII protein profile, with two prominent adjacent bands of molecular weight around 60 kDa (Figure 5).

## Discussion

Crustaceans have been used as a biological model by many research groups in neurobiology, because of the similarities in the cellular machinery in the nervous system of crustaceans and vertebrates, suggesting that common strategies have evolved across animal phyla [29], [30], [31], [32]. In this study we showed for the first time the location of CaMKII in the prawn eyestalk and brain areas directly involved with the processing of sensory stimuli [28]. Therefore, our study concords with previous findings that this molecule plays analogous roles in the crustacean brain to those in the vertebrate central nervous system [10], [11], [12], [27], [33].

Different stimuli activate neurons that project to specialized sensory brain areas. The eyestalk contains brain regions that receive intensity-specific stimuli from the environment. Regarding light reception, different response patterns are processed in the eyestalk ganglia, such as perception of light and dark, depth, dimensions, and spatial location [34], [35], [36], [37]. In general, the visual sensory stimuli are converted into motor responses and eventually translated into behaviors [38]. In a previous report, we demonstrated in freshwater prawns and marine shrimps that the OL is responsible for converting sensory stimuli into motor information [19]. Here, we showed that CaMKII is expressed in the same sites as FMRFamide (which was previously used to control for the labeling of neuronal elements in crustaceans [24], [25]) and one postsynaptic glutamate receptor in the sensory structures. This observation indicates that the synaptic areas in these regions may also be related to sensory-motor processing, and contributes to the understanding of the complex functions in the different brain zones of crustaceans.

Western blotting from the brain homogenates of *M. acanthurus* resulted in two molecular bands, very close to each other, for CaMKII. These bands possibly correspond to isoforms of this kinase, since gene regulation of CaMKII is unclear in invertebrates, and studies [6], [15] have demonstrated the existence of isoforms produced by alternative splicing. The production of this variety of isoforms can allow the phosphorylation of different substrates by the same cell. The activity of CaMKII on the postsynaptic sites is sufficient to augment the release of the appropriate presynaptic neurotransmitter for the functioning of the visual input [7], [39]. Interestingly, in the photoreceptors of *M. acanthurus* we observed a strong labeling for CaMKII and GluA1, which therefore probably have the role of sustaining the excitatory synapse.

Regarding the OL, the projections of the sensory neurons of the antennules reach the cap [25] and are projected to the subcap. This region in *M. acanthurus* was intensely labeled for CaMKII and FMRFamide; according to [40], at the neuromuscular junction, CaMKII modulates the synaptic potential of DRNFLRFamide, a neurotransmitter of the same chemical group as FMRFamide. Since we found FMRFamide labeling in the antennal neuropil and in the tegumentary neuropil - which function as motor nuclei, as formerly described by [41] using Golgi staining and methylene blue - this is in line with [42] who claimed that FMRFamide activates a complex sequence of intracellular signaling events that augment the release of the transmitter from the synaptic terminals of motor neurons.

Although the literature refers to the importance of CaMKII in crustaceans, as outlined above, no information exists on the location of CaMKII *in situ* in the nervous system of crustaceans. Calmodulins and CaMKII have been identified in other crustaceans only by biochemical approaches; however, to our knowledge, no information regarding their location was given. [9] described the purification of calmodulin and some of its properties from the crayfish *Orconectes limosus*; [43] isolated and purified calmodulin from the shrimp *Crangon crangon*; and [11] characterized the CaMKII activity in nervous tissue of the lobster *Panulirus interruptus*. The novelty of our results is precisely the identification of those cells and regions of the cerebral ganglia where the expression of CaMKII is enhanced, thus contributing to the understanding of its role in the neural processing of environmental information in crustaceans.

## References

[1] McNeil RB, Colbran RJ (1995) Interaction of autophosphorylated Ca/calmodulin-dependent protein kinase II with neuronal cytoskeletal proteins. J Biol Chem 270: 10043–10049. 7730306 10.1074/jbc.270.17.10043

[2] Hawkins RD, Kandel ER, Bailey CH (2006) Molecular mechanisms of memory storage in Aplysia. Biol Bull 210: 174–191. 16801493 10.2307/4134556

[3] Coultrap SJ, Bayer KU (2012) CaMKII regulation in information processing and storage. Trends in Neurosciences 35: 607–618. 22717267 10.1016/j.tins.2012.05.003 PMC3461103

[4] Soderling TR (2000) CaM-kinases: modulators of synaptic plasticity. Curr Opin Neurobiol 10: 375–380. 10851169 10.1016/s0959-4388(00)00090-8

[5] Rongo C, Kaplan JM (1999) CaMKII regulates the density of central glutamatergic synapses in vivo. Nature 402: 195–199. 10647013 10.1038/46065

[6] GuptaRoy B, Beckingham K, Griffith LC (1996) Functional Diversity of Alternatively Spliced Isoforms of Drosophila Ca21/Calmodulin-dependent Protein Kinase II: A role for the variable domain in activation. The Journal of Biological Chemistry 271: 19846–19851. 8702694 10.1074/jbc.271.33.19846

[7] Haghighi AP, McCabe BD, Fetter RD, Palmer JE, Hom S, et al (2003) Retrograde control of synaptic transmission by postsynaptic CaMKII at the Drosophila neuromuscular junction. Neuron 39: 255–267. 12873383 10.1016/s0896-6273(03)00427-6

[8] Burkert P, Duch C (2006) Developmental changes of CaMKII localization, activity and function during postembryonic CNS remodelling in Manduca sexta. Eur J Neurosci 23: 335–349. 16420442 10.1111/j.1460-9568.2005.04562.x

[9] Hergenhahn HG, Kegel G, Sedlmeier D (1984) Ca2+-binding proteins in crayfish abdominal muscle. Evidence for a calmodulin lacking trimethyllysine. Biochim Biophys Acta 787: 196–203. 6329304 10.1016/0167-4838(84)90080-3

[10] Chang BH, Mukherji S, Soderling TR (1998) Characterization of a calmodulin kinase II inhibitor protein in brain. Proc Natl Acad Sci U S A 95: 10890–10895. 9724800 10.1073/pnas.95.18.10890 PMC27991

[11] Withers MD, Kennedy MB, Marder E, Griffith LC (1998) Characterization of calcium/calmodulin-dependent protein kinase II activity in the nervous system of the lobster, Panulirus interruptus. Invert Neurosci 3: 335–345. 10212401 10.1007/BF02577693

[12] Chen N, Furuya S, Doi H, Hashimoto Y, Kudo Y, et al (2003) Ganglioside/calmodulin kinase II signal inducing cdc42-mediated neuronal actin reorganization. Neuroscience 120: 163–176. 12849750 10.1016/s0306-4522(03)00259-8

[13] Hudmon A, Schulman H (2002) Neuronal CA2+/calmodulindependent protein kinase II: the role of structure and autoregulation in cellular function. Annu Rev Biochem 71: 473–510. 12045104 10.1146/annurev.biochem.71.110601.135410

[14] Hanson PI, Schulman H (1992) Neuronal Ca/calmodulin-dependent protein kinases. Ann Rev Biochem 61: 559–601. 1323238 10.1146/annurev.bi.61.070192.003015

[15] GuptaRoy B, Marwaha N, Pla M, Wang Z, Nelson HB, et al (2000) Alternative splicing of Drosophila calcium/calmodulin-dependent protein kinase II regulates substrate specificity and activation. Brain Res Mol Brain Res 80: 26–34. 11039726 10.1016/s0169-328x(00)00115-7

[16] Cho KO, Wall JB, Pugh PC, Ito M, Mueller SA, et al (1991) The alpha subunit of type II Ca2+/calmodulin-dependent protein kinase is highly conserved in Drosophila. Neuron 7: 439–450. 1910789 10.1016/0896-6273(91)90296-c

[17] Sandeman DC, Scholtz G (1995) Ground plans, evolutionary changes and homologies in decapod crustacean brains. In: Breidbach O, Kutsch W, editors. The nervous systems of invertebrates: an evolutionary and comparative approach. Basel: Birkhäuser. 329–347.

[18] Langworthy K, Helluy S, Benton J, Beltz B (1997) Amines and peptides in the brain of the American lobster: immunocytochemical localization patterns and implications for brain function. Cell Tissue Res 288: 191–206. 9042786 10.1007/s004410050806

[19] Ammar D, Nazari EM, Rauh Muller YM, Allodi S (2008) New insights on the olfactory lobe of decapod crustaceans. Brain Behav Evol 72: 27–36. 18560210 10.1159/000139459

[20] Schmidt M (1997) Distribution of presumptive chemosensory afferents with FMRFamide- or substance P-like immunoreactivity in decapod crustaceans. Brain Res 746: 71–84. 9037486 10.1016/s0006-8993(96)01187-0

[21] Horner AJ, Weissburg MJ, Derby CD (2004) Dual antennular chemosensory pathways can mediate orientation by Caribbean spiny lobsters in naturalistic flow conditions. J Exp Biol 207: 3785–3796. 15371486 10.1242/jeb.01200

[22] Sullivan JM, Beltz BS (2004) Evolutionary changes in the olfactory projection neuron pathways of eumalacostracan crustaceans. J Comp Neurol 470: 25–38. 14755523 10.1002/cne.11026

[23] Schmidt M, Derby CD (2005) Non-olfactory chemoreceptors in asymmetric setae activate antennular grooming behavior in the Caribbean spiny lobster Panulirus argus. J Exp Biol 208: 233–248. 15634843 10.1242/jeb.01357

[24] Allodi S, Bressan CM, Carvalho SL, Cavalcante LA (2006) Regionally specific distribution of the binding of anti-glutamine synthetase and anti-S100 antibodies and of Datura stramonium lectin in glial domains of the optic lobe of the giant prawn. Glia 53: 612–620. 16435368 10.1002/glia.20317

[25] Johansson KUI, Hallberg E (1992) The organization of the olfactory lobes in Euphausiacea and Mysidacea (Crustacea, Malacostraca). Zoomorphology 112: 81–89.

[26] Sombke A, Harzsch S, Hansson BS (2010) organization of deutocerebral neuropils and olfactory behavior in the centipede Scutigera coleoptrata (Linnaeus, 1758) (Myriapoda: Chilopoda). Chem Senses 36: 43–61. 20962283 10.1093/chemse/bjq096

[27] Peterson GL (1983) Determination of total protein. Methods Enzymol 91: 95–119. 6855607 10.1016/s0076-6879(83)91014-5

[28] Schachtner J, Schmidt M, Homberg U (2005) Organization and evolutionary trends of primary olfactory brain centers in Tetraconata (Crustacea+Hexapoda). Arthropod Structure & Development 34: 257–299.

[29] Harzsch S, Dawirs RR (1993) On the morphology of the central nervous system in larval stages of Carcinus maenas L. (Decapoda, Brachyura). Helgoland Mar Res 47: 61–79.

[30] Sandeman DC, Scholtz G, Sandeman RE (1993 ) Brain evolution in decapod Crustacea. J Exp Zool 265: 112–133.

[31] Derby CD, Sorensen PW (2008) Neural processing, perception, and behavioral responses to natural chemical stimuli by fish and crustaceans. J Chem Ecol 34: 898–914. 18521679 10.1007/s10886-008-9489-0

[32] Zhang Y, Allodi S, Sandeman DC, Beltz BS (2009) Adult neurogenesis in the crayfish brain: proliferation, migration, and possible origin of precursor cells. Dev Neurobiol 69: 415–436. 19294644 10.1002/dneu.20717 PMC4479499

[33] Fingerman M, Hanumante MM, Kulkarni GK, Ikeda R, Vacca LL (1985) Localization of substance P-like, leucine-enkephalin-like, methionine-enkephalin-like, and FMRFamide-like immunoreactivity in the eyestalk of the fiddler crab, Uca pugilator. Cell Tissue Res 241: 473–477. 2411411 10.1007/BF00214565

[34] Melzer RR, Diersch R, Nicastro D, U S (1997) Compound eye evolution: highly conserved retinula and cone cell patterns indicate a common origin of the insect and crustacean ommatidium. Naturwissenschaften 84: 542–544.

[35] Meyer-Rochow VB (2001) The Crustacean eye: dark/light adaptation, polarization sensitivity, flicker fusion frequency, and photoreceptor damage. Zool Sci 18: 1175–1197. 11911074 10.2108/zsj.18.1175

[36] Arendt D (2003) Evolution of eyes and photoreceptor cell types. Int J Dev Biol 47: 563–571. 14756332

[37] Kleinlogel S, Marshall NJ, Horwood JM, Land MF (2003) Neuroarchitecture of the color and polarization vision system of the stomatopod Haptosquilla. J Comp Neurol 467: 326–342. 14608597 10.1002/cne.10922

[38] Joiner Ml A, Griffith LC (1997) CaM kinase II and visual input modulate memory formation in the neuronal circuit controlling courtship conditioning. J Neurosci 17: 9384–9391. 9364084 10.1523/JNEUROSCI.17-23-09384.1997 PMC6573606

[39] Kazama H, Morimoto-Tanifuji T, Nose A (2003) Postsynaptic activation of calcium/calmodulin-dependent protein kinase II promotes coordinated pre- and postsynaptic maturation of Drosophila neuromuscular junctions. 117: 615–625. 10.1016/s0306-4522(02)00923-5 12617966

[40] Noronha KF, Mercier AJ (1995) A role for calcium/calmodulin-dependent protein kinase in mediating synaptic modulation by a neuropeptide. Brain Res 673: 70–74. 7757481 10.1016/0006-8993(94)01396-y

[41] Tsvileneva V, Titova VA (1985) On the brain structures of decapods. Zool Jahrb Anat 113: 217–266.

[42] Mercier AJ, Badhwar A, Weston AD, Klose M (2002) Intracellular signals that mediate synaptic modulation by a FMRFamide-like neuropeptide in crayfish. In: Wiese K, editor. The Crustacean Nervous System. Heidelberg: Springer. 49–62.

[43] Michael RH, Pipkorn R, Willig A, Jaros PP (1992) Isolation and purification of calmodulin from the shrimp, Crangon crangon. Life Sci 51: 1881–1889. 1333028 10.1016/0024-3205(92)90040-v

